# Transformation of hydrogen titanate nanoribbons to TiO_2_ nanoribbons and the influence of the transformation strategies on the photocatalytic performance

**DOI:** 10.3762/bjnano.6.86

**Published:** 2015-03-27

**Authors:** Melita Rutar, Nejc Rozman, Matej Pregelj, Carla Bittencourt, Romana Cerc Korošec, Andrijana Sever Škapin, Aleš Mrzel, Srečo D Škapin, Polona Umek

**Affiliations:** 1 Jožef Stefan Institute, Jamova cesta 39, SI-1000 Ljubljana, Slovenia; 2 Jožef Stefan International Postgraduate School, Jamova cesta 39, SI-1000 Ljubljana, SIovenia; 3 Slovenian National Building and Civil Engineering Institute, Dimičeva 12, SI-1000 Ljubljana, Slovenia; 4 Chimie des Interactions Plasma Surface, CIRMAP, University of Mons, 23 Place du Parc, B-7000 Mons, Belgium; 5 Faculty of Chemistry and Chemical Technology, University of Ljubljana, Aškerčeva cesta 5, SI-1000 Ljubljana, Slovenia

**Keywords:** doping, nanoribbon, photocatalytic performance, titanate, titanium dioxide (TiO_2_), transformation

## Abstract

The influence of the reaction conditions during the transformation of hydrogen titanate nanoribbons to TiO_2_ nanoribbons on the phase composition, the morphology, the appearance of the nanoribbon surfaces and their optical properties was investigated. The transformations were performed (i) through a heat treatment in oxidative and reductive atmospheres in the temperature range of 400–650 °C, (ii) through a hydrothermal treatment in neutral and basic environments at 160 °C, and (iii) through a microwave-assisted hydrothermal treatment in a neutral environment at 200 °C. Scanning electron microscopy investigations showed that the hydrothermal processing significantly affected the nanoribbon surfaces, which became rougher, while the transformations based on calcination in either oxidative or reductive atmospheres had no effect on the morphology or on the surface appearance of the nanoribbons. The transformations performed in the reductive atmosphere, an NH_3_(g)/Ar(g) flow, and in the ammonia solution led to nitrogen doping. The nitrogen content increased with an increasing calcination temperature, as was determined by X-ray photoelectron spectroscopy. According to electron paramagnetic resonance measurements the calcination in the reductive atmosphere also resulted in a partial reduction of Ti^4+^ to Ti^3+^. The photocatalytic performance of the derived TiO_2_ NRs was estimated on the basis of the photocatalytic oxidation of isopropanol. After calcinating in air, the photocatalytic performance of the investigated TiO_2_ NRs increased with an increased content of anatase. In contrast, the photocatalytic performance of the N-doped TiO_2_ NRs showed no dependence on the calcination temperature. An additional comparison showed that the N-doping significantly suppressed the photocatalytic performance of the TiO_2_ NRs, i.e., by 3 to almost 10 times, in comparison with the TiO_2_ NRs derived by calcination in air. On the other hand, the photocatalytic performance of the hydrothermally derived TiO_2_ NRs was additionally improved by a subsequent heat treatment in air.

## Introduction

Titanium dioxide (TiO_2_) is a technologically important material due to its remarkable combination of properties, its chemical stability and nontoxicity [1–2]. In particular, a great deal of attention has paid to the research and exploitation of its catalytic and photocatalytic properties in a variety of applications, ranging from the environmental to energy applications [3–5]. The key factors that determine the photocatalytic efficiency of TiO_2_ are its phase composition, specific surface area, doping, particle size and morphology [5–7]. This means that the photocatalytic efficiency of TiO_2_ can be significantly altered by the proper selection of the TiO_2_ precursor, the reaction conditions and/or any post-treatment conditions. Typically, TiO_2_ nanomaterials are synthesized by using the sol–gel method applying various titanium(IV) alkoxides or TiCl_4_ as the precursors [2]. The transformation of layered titanates to TiO_2_ through ion exchange and subsequent dehydration represents an alternative pathway [8] to the well-established methods for the preparation of TiO_2_. Since the first report on the synthesis of titanate nanotubes by Kasuga et al. [9], this pathway has become more interesting mostly due to its high synthesis yield and the possibility of using sodium titanate nanotubes as a precursor for the preparation of TiO_2_ nanotubes.

Titanates, with the formula A_2_Ti*_n_*O_2_*_n_*_+1_, form a variety of layered structures for 3 ≤ *n* ≤ 6 in which the alkali cations (A) occupy the interlayer space. Thus, located alkali cations are mobile and are easily exchanged by protons. The resulting hydrogen titanates have poor thermal stability and can be easily transformed to TiO_2_ upon heating. This makes these alkali titanates an attractive precursor for the preparation of different TiO_2_ polymorphs. They can be synthesized through a solid-state reaction from the corresponding metal carbonates and TiO_2_ or hydrothermally from alkali hydroxide and TiO_2_ [10]. The latter method is of special interest because it enables the formation of one-dimensional (1D) alkali titanate nanoparticles in large quantities. Moreover, the control of the morphology can be achieved by a simple regulation of the reaction temperature or by the selection of an alkali hydroxide [7,11–12]. Thus alkali titanate 1D nanostructures such as nanotubes [9,12], nanowires [13], nanofibers or nanoribbons [12] (NR) morphologies can be obtained.

Transformations from the layered titanate structure to TiO_2_-B and then to the anatase structure (H_2_Ti_3_O_7_ → TiO_2_-B → anatase) are considered to be topotactic reactions [14–15] and due to the structural similarities between these three structures, the morphology is preserved during these transformations. In general, the nanotube morphology is more desirable because of its high specific surface area. However, due to their denser structure, nanoribbons and nanowires are more appropriate for subsequent processing, in terms of preserving their morphology. The transformation of hydrogen titanate nanoribbons (HTiNRs) occurs under mild reaction conditions, either by calcination in different atmospheres or under wet-chemical conditions. The thermal treatment of HTiNRs at low calcination temperatures (ca. 400 °C) results in the formation of a metastable TiO_2_-B phase [8,16] which at higher temperatures transforms to anatase [17–19]. Transformations conducted under reflux or hydrothermal conditions in neutral and acidic environment affect the surface of the nanoribbons [14,20–21].

With the careful selection of the transformation strategy and the processing parameters it is possible to tailor the surface doping, the TiO_2_ phase composition and/or the specific surface area of the TiO_2_ products. For instance, transformations in an NH_3_ atmosphere result in N-doped TiO_2_ [22] in which the nitrogen atoms occupy substitutional and interstitial sites in the TiO_2_ and thus affect the photocatalytic activity of titania for reactions performed under visible-light irradiation [3,5,23–24]. On the other hand, transformation under wet-chemical or hydrothermal conditions strongly alters the nanoribbon surfaces and as a result the specific surface area increases [14,20]. Interestingly, a change in the reaction medium from neutral to acidic allows the transformation to occur under almost ambient conditions. Furthermore, under these conditions, TiO_2_/titanate hybrid nanostructures are formed [14,21].

In the present work, we used hydrogen titanate nanoribbons as a precursor for the preparation of TiO_2_ NRs. A systematic study of the impact of the transformation conditions on the photocatalytic performance of derived TiO_2_ NRs was undertaken. The TiO_2_ NRs were prepared by different post treatments of the HTiNRs: (i) calcination in air and in a flow of NH_3_(g)/Ar(g), (ii) convective heating under hydrothermal conditions in deionized water and in a 0.5 M NH_3_(aq) solution, and (iii) microwave-accelerated heating (microwave-assisted hydrothermal transformation). For the first time, to the best of our knowledge, a hydrothermal transformation conducted in NH_3_(aq) is reported. The transformations performed in the NH_3_(g)/Ar(g) flow or NH_3_(aq) led to N doping. The obtained samples were characterized with a variety of analytical techniques, with the aim being to evaluate the TiO_2_ phase composition, the morphology, the content of nitrogen, the chemical state of the nitrogen, the specific surface area and the presence of Ti^3+^. The photocatalytic performances of the prepared TiO_2_ NRs were evaluated by monitoring the oxidation of isopropanol to acetone in a solid gas reactor.

## Results and Discussion

In this study we used hydrogen titanate nanoribbons (HTiNRs) as a precursor for the preparation of TiO_2_ nanoribbons [17,25]. A typical scanning electron microscopy (SEM) image of the precursor NRs (Figure S1, Supporting Information File 1) reveals their smooth and clean surfaces, as well as a high nanoribbon content. Their widths were found to be in the range of 30–250 nm, while the average length varied from 1 to 4 μm [26]. In some cases the lengths of the individual nanoribbons can reach up to 10 μm. The powder X-ray diffraction (XRD) pattern of the precursor NRs (Figure S2, Supporting Information File 1) matches those of hydrogen titanates (H_2_Ti_3_O_7_) reported in the literature [17,27–28]. In addition, the chemical analysis, performed by using energy dispersive X-ray spectrometry (EDX), showed that the sodium content in the HTiNRs is below 0.1 wt %.

H_2_Ti_3_O_7_ is thermally very unstable and, therefore, can be easily converted to TiO_2_. Depending on the nanoparticle size and/or morphology this transition can occur at temperatures even below 350 °C [11,27]. A thermogravimetric (TG) curve of the precursor nanoribbons with an accompanying differential scanning calorimetry (DSC) curve (Figure S3, Supporting Information File 1) shows that the dehydration process of more tightly bound water (100–250 °C) is accompanied by the first structural changes (180–230 °C). In our case, the transformation from H_2_Ti_3_O_7_ to TiO_2_ was carried out (i) through a heat treatment in static air and a dynamic NH_3_(g)/Ar(g) atmosphere, (ii) through a conventional heating under hydrothermal conditions in deionized water and in a basic environment, and (iii) through microwave-assisted hydrothermal conditions in deionized water. The exact experimental conditions and the phase compositions of the derived TiO_2_ NRs are summarized in Table 1.

**Table 1:** Experimental conditions (reaction temperature (*T*), reaction time (*t*) and heating rate (Δ*T*/Δ*t*)) for the transformation of hydrogen titanate nanoribbons to TiO_2_ nanoribbons in different environments and the phase composition of the samples.

sample	transformation environment	*T* (°C)	*t* (h)	Δ*T*/Δ*t* (°C·min^−1^)	phase composition

**TO-400**	static air	400	6	1	TiO_2_-B
**TO-580**	static air	580	6	1	anatase + TiO_2_-B
**TO-650**	static air	650	6	1	anatase

**TN-400**	NH_3_(g)/Ar(g) flow	400	6	7.2	TiO_2_-B
**TN-580**	NH_3_(g)/Ar(g) flow	580	6	7.2	anatase + TiO_2_-B
**TN-650**	NH_3_(g)/Ar(g) flow	650	6	7.2	anatase + TiO_2_-B

**CH-W**^a^	deionized water	160	10	4	anatase
**CH-N**^a^	0.5 M NH_3_(aq)	160	24	4	anatase

**MW-W**^b^	deionized water	200	2	as fast as possible	anatase

**MW-W+TO**	1^st^ deionized water, 2^nd^ static air	200 480	2 6	as fast as possible 1	anatase

**CH-W+TN**	1^st^ deionized water, 2^nd^ NH_3_(g)/Ar(g) flow	200 400	10 6	4 7.2	anatase

^a^hydrothermal reactor; ^b^microwave reactor.

### Structural determination of TiO_2_ polymorphs

**Figure 1:**
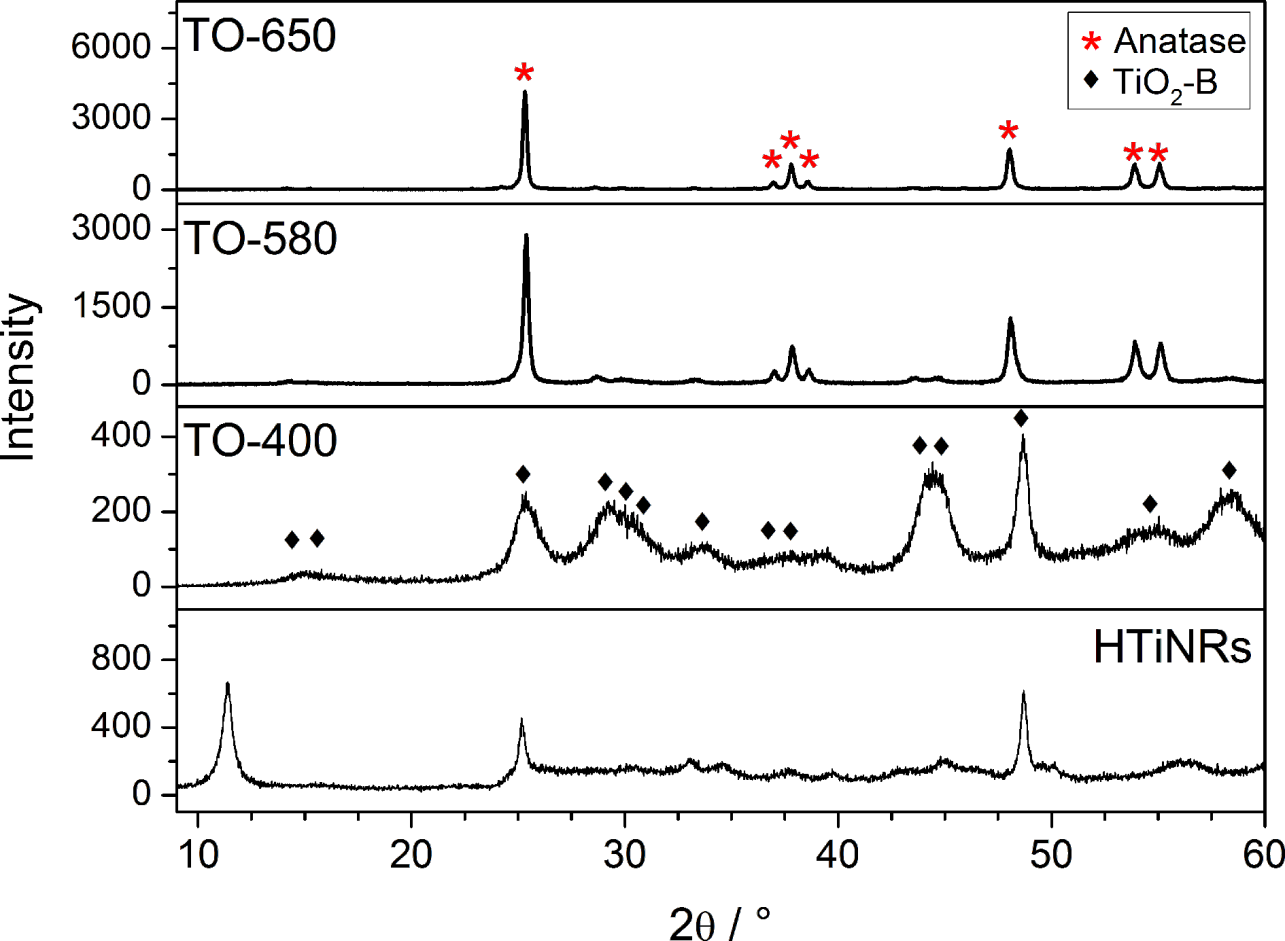
XRD patterns of precursor hydrogen titanate nanoribbons and TiO_2_ nanoribbons derived from hydrogen titanate nanoribbons resulting from a heat treatment in air at 400, 580 and 650 °C.

Figure 1 shows the powder XRD patterns of the TiO_2_ nanoribbons resulting from the calcination of HTiNRs in air at 400, 580 and 650 °C. Upon heating at 400 °C the HTiNRs completely transformed to TiO_2_-B (**TO-400**, JCPDS No. 35-0088). With an increase in the calcination temperature (580 °C) new peaks emerged in the XRD pattern of **TO-580** corresponding to anatase. The transformation from TiO_2_-B to anatase was completed at 650 °C. The diffractogram of **TO-650** corresponds to anatase (JCPDS No. 86-1157).

**Figure 2:**
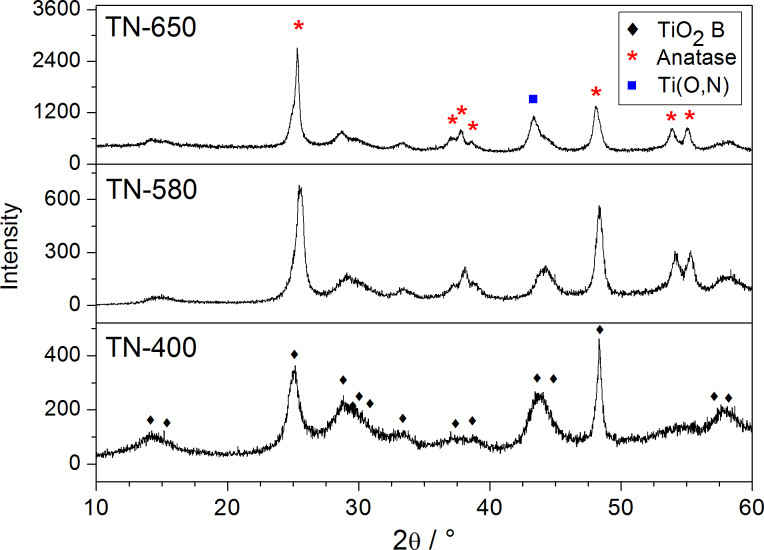
XRD patterns of TiO_2_ nanoribbons derived from hydrogen titanate nanoribbons resulting from a heat treatment in a NH_3_(g)/Ar(g) flow at 400, 580 and 650 °C.

As in the case of the calcination in air, a similar sequence of transformations in the same temperature range, is observed from the powder XRD patterns of the TiO_2_ NRs obtained by calcination in the NH_3_(g)/Ar(g) flow (Figure 2). The diffractogram of the sample calcined at 400 °C (**TN-400**) corresponds to TiO_2_-B, while, according to the diffractograms, the samples heated at 580 and 650 °C (**TN-580** and **TN-650**) are mixtures of TiO_2_-B and anatase. A direct comparison of the XRD patterns of the samples calcined in air (Figure 1) and in the NH_3_(g)/Ar(g) flow (Figure 2) at the same temperatures gives the impression that the transformation rate from TiO_2_-B to anatase is slower in the reductive atmosphere than in static air. This difference arises from the difference in the temperature ramp rates, which were significantly higher for the experiments performed in the NH_3_(g)/Ar(g) flow (Table 1). Interestingly, a new peak appears in the XRD pattern of **TN-650** that corresponds to cubic titanium oxynitride [29].

**Figure 3:**
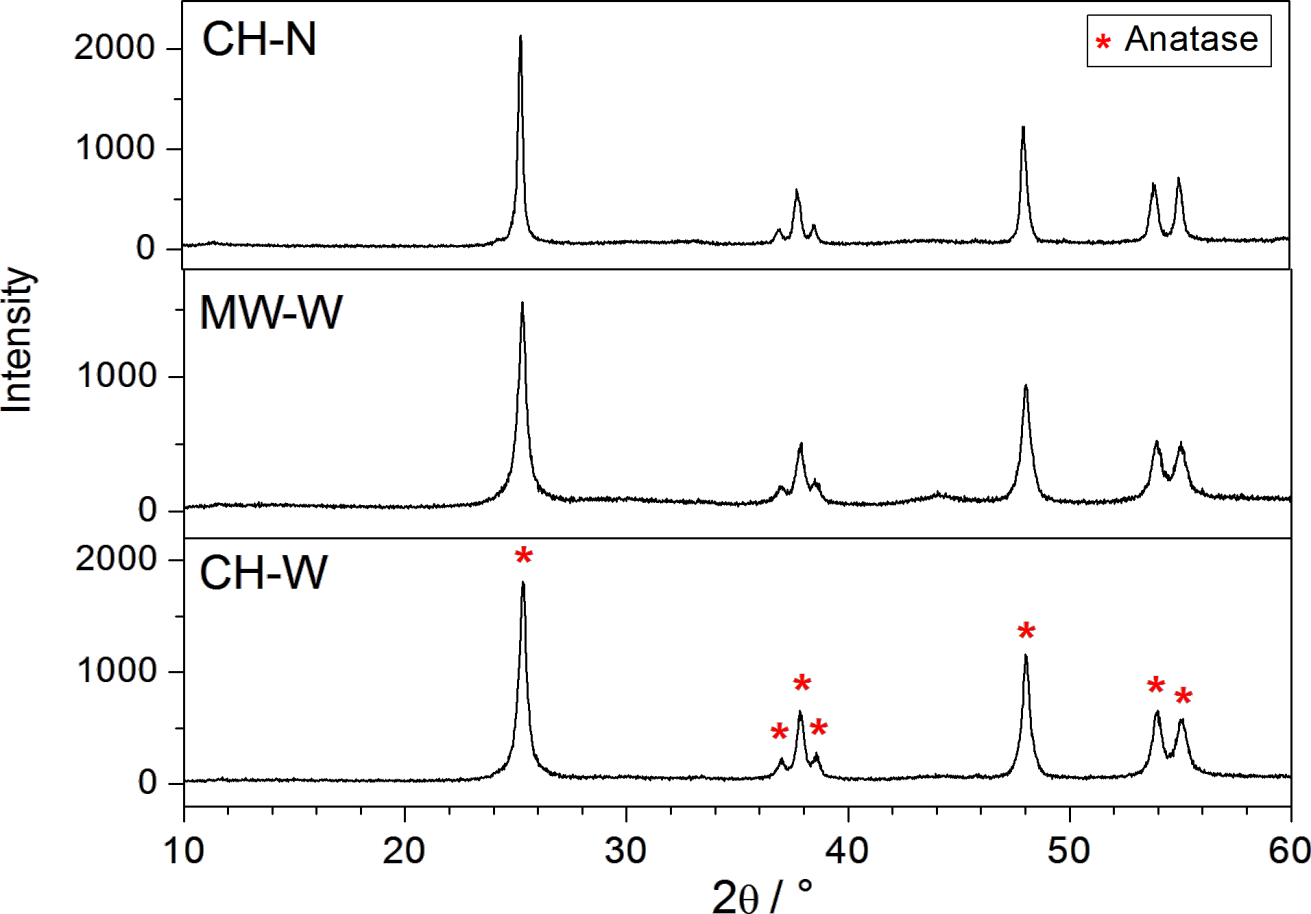
XRD patterns of TiO_2_ nanoribbons derived from hydrogen titanate nanoribbons in water in a hydrothermal reactor (**CH-W**), a microwave reactor (**MW-W**), and in 0.5 M NH_3_(aq) in a hydrothermal reactor (**CH-N**).

Next, the influence of the pH value of the reaction medium on the reaction times of the hydrothermal and microwave-assisted hydrothermal transformations was also investigated. According to the powder XRD patterns (Figure 3), the processing time required for complete conversion in the hydrothermal reactor, in deionized water at 160 °C (**CH-W**), was 10 h. In contrast, the transformation in 0.5 M NH_3_(aq) (**CH-N**) was only completed after 24 h. In addition, the experiments in ammonia solutions with higher molarities did not produce any TiO_2_.

Aiming to shorten the time needed for the transformation of HTiNRs into TiO_2_ NRs, the reaction was also carried out in a microwave reactor. The powder XRD pattern of **MW-W** (Figure 3) shows that this transformation proceeds significantly faster (2 h), although at a slightly higher temperature (200 °C) than with **CH-W**. Additionally, when the transformations were performed hydrothermally in a conventional autoclave or in a microwave reactor, no TiO_2_-B was observed as an intermediate phase, even at temperatures lower than 160 °C and reaction times shorter than 2 h.

A comparison of the powder XRD pattern of the calcined TiO_2_ NRs (**TO-650**) with the XRD patterns of the hydrothermally derived TiO_2_ NRs (**CH-W**) and the sample produced under microwave irradiation (**MW-W**) shows a lower crystallinity in the latter two cases. The additional annealing of **CH-W** and **MW-W** in the NH_3_(g)Ar(g) flow and air, respectively, resulted in an improved crystallinity of derived TiO_2_ NRs (**CH-W+TN** and **MW-W+TO**, Figure S4, Supporting Information File 1).

### Morphology characterization

**Figure 4:**
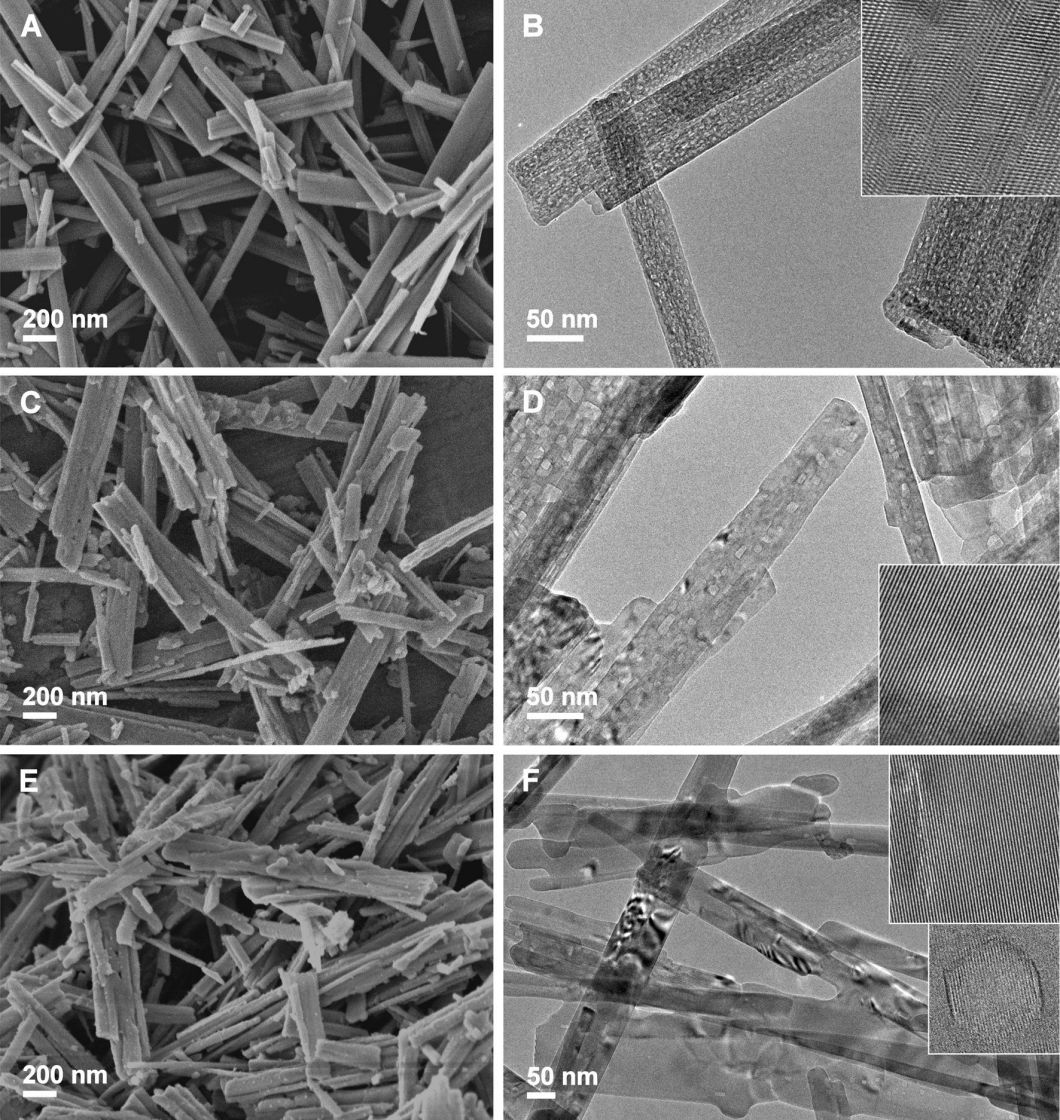
SEM and TEM images of TiO_2_ nanoribbons derived from hydrogen titanate nanoribbon precursors by heat treatment in air at 400 °C (A and B), 580 °C (C and D), and 650 °C (E and F). The insets in figures B, D and F indicate the crystalline nature of the nanoribbons.

In the next step, SEM and TEM were used to monitor the morphological evolution due to the processing conditions and to check the surfaces of the formed TiO_2_ NRs. In general, from the SEM images of the TiO_2_ NRs resulting from the calcination in air (Figure 4) it is clear that the nanoribbon morphology is preserved up to 650 °C. However, a detailed TEM investigation (Figure 4) revealed that small changes in the shapes of the nanoribbons already occurred at 580 °C (**TO-580**). The nanoribbon edges became rounder due to partial sintering. As expected, these changes are more obvious in the **TO-650** sample.

The TEM images presented in Figure 4, in addition to the slight changes in the shape of the nanoribbons, reveal the impact of the calcination temperature on the surface of the nanoribbons. The conversion from hydrogen titanate to TiO_2_-B NRs resulted in the formation of pores [17] with diameters in the range of 2–10 nm. This is a direct consequence of the interlayered OH groups leaving the structure [30]. Not surprisingly, the pore formation, affecting the crystallinity of the samples, is also reflected in the width of the XRD peaks (Figure 1A) [17]. With an increased calcination temperature the number of pores decreases and, consequently the nanoribons surfaces become smoother.

The SEM images of the TiO_2_ nanoribbons derived thermally in the NH_3_(g)/Ar(g) flow at 400, 580 and 650 °C (Figure S5, Supporting Information File 1) again reveal that the nanoribbon morphology is completely preserved up to 650 °C. The TEM images (Figure S5, Supporting Information File 1), on the other hand, show that porous structure of the nanoribbons is not reduced at higher calcination temperatures, as in the case with the TiO_2_ NRs calcined in air (Figure 4). Most likely, this is due to relatively high TiO_2_-B content of in **TN-650,** as can be concluded from the X-ray diffractogram (Figure 2).

**Figure 5:**
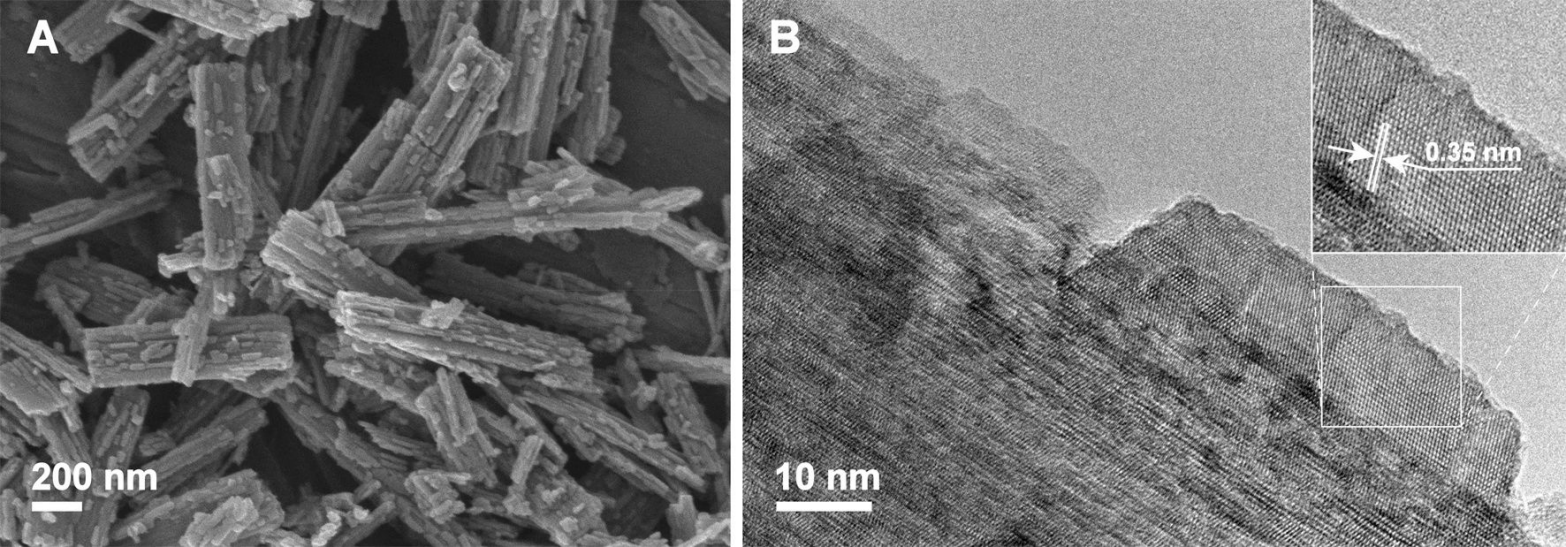
A) SEM and B) TEM images of TiO_2_ nanoribbons derived from hydrogen titanate nanoribbons after treatment in a microwave reactor at 200 °C for 2 h in deionized water (**MW-W**). The inset to B: the measured interplanar spacing corresponds to a *d* spacing of 0.35 nm and is consistent with the (101) planes of anatase.

**Figure 6:**
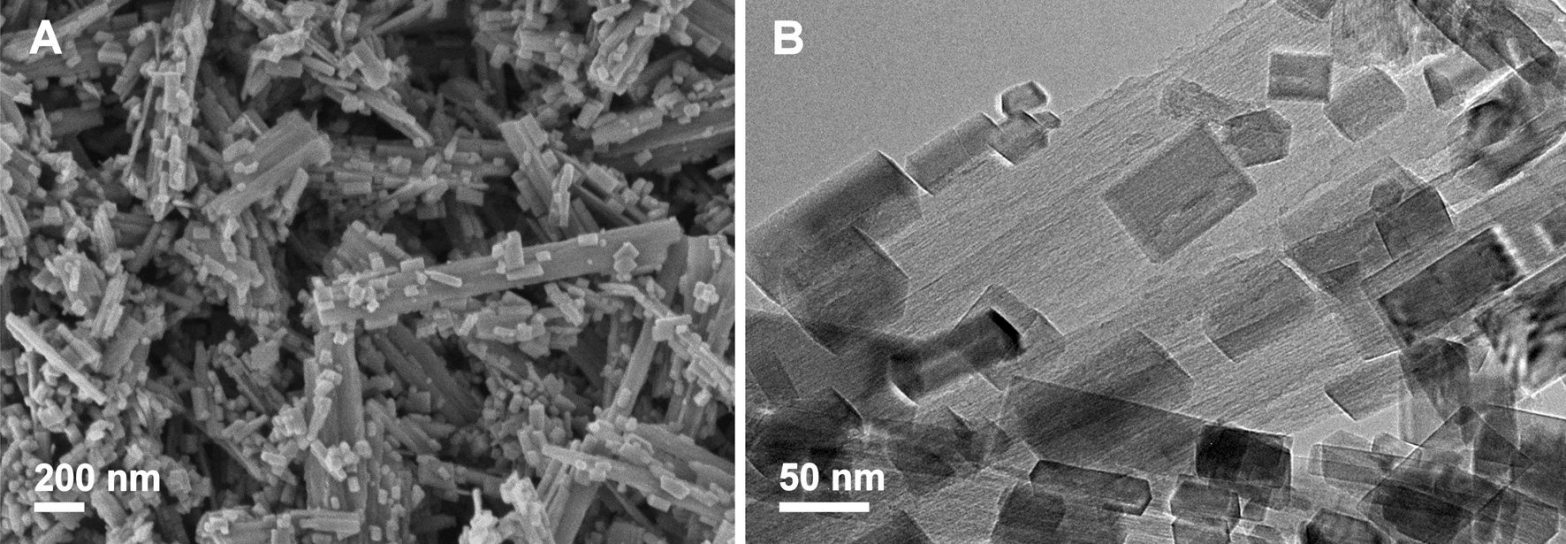
A) SEM and B) TEM images of N-doped TiO_2_ nanoribbons derived from hydrogen titanate nanoribbons by hydrothermal treatment in 0.5 M NH_3_(aq) at 160 °C for 10 h.

The hydrothermal processing of HTiNRs by convective heating (**CH-W**) or in microwave-accelerated heating (**MW-W**) significantly affected the surfaces of the formed TiO_2_ NRs. In Figure 5 SEM and TEM images of **MW-W** are shown. From a comparison with the SEM images of the anatase NRs obtained after thermal (Figure 4 and Figure S5 (Supporting Information File 1)) and hydrothermal processing (Figure 5A) it is apparent that the hydrothermal conditions are more severe and lead to rougher nanoribbon surfaces. An investigation with high resolution transmission electron microscopy (HRTEM) (Figure 5B) revealed that nanoparticles with a trapezoidal shape were formed on the surfaces of the nanoribbons. The observed effect was the same for **CH-W**. Further, we observed that a change of the reaction environment, i.e., deionized water was exchanged with 0.5 M NH_3_(aq), had a significant impact on the shape of these nanoparticles. In 0.5 M NH_3_(aq) the formed nanostructures have rectangular or elongated hexagonal shapes (Figure 6A and 6B).

When the transformation proceeded hydrothermally (**CH-W**, **CH-N** and **MW-W**), the morphology of the nanoribbons is preserved due to an in situ rearrangement of the structural TiO_6_ units while small crystallites that cover the nanoribbon surfaces (Figure 5 and Figure 6) are formed through a dissolution–recrystallization process, as suggested by Zhu et al. [14]. The shape of these crystallites strongly depends on the pH of the reaction medium since different ions act as capping agents [31]. Published results [14,20–21] suggest that the stability of HTiNRs in aqueous suspensions decreases with the decreasing pH. This is in the agreement with our results since the transformation occurred only with 0.5 M NH_3_(aq) and not in ammonia solutions with larger molarities. Also the time needed for the complete transformation in the basic environment was significantly longer (24 h, **CH-N**) compared with 10 h (**CH-W**) in deionized water.

### Determination of the nitrogen content

An X-ray photoelectron spectroscopy (XPS) study was conducted to determine the nitrogen content and to investigate its chemical state in the N-doped TiO_2_ NRs. The results are shown in Table 2. As expected, the nitrogen content increases with a higher calcination temperature; i.e., from 0.8 wt % (1.5 atom %) in **TN-400** to 2.5 wt % (5.0 atom %) in **TN-650**. Interestingly, the lowest nitrogen content of 0.6 wt % (0.8 atom %) was obtained when the doping was performed in a 0.5 M NH_3_(aq) solution (**CH-N**). In addition, the thermal treatment of HTiNRs in an NH_3_(g)/Ar(g) flow affected the color of resulting N-doped TiO_2_ NRs (Table 2), which changed from white (HTiNRs) to pale yellow, yellowish green and grey when calcined at 400, 580 and 650 °C, respectively.

**Table 2:** Nitrogen content, specific surface area (*S*_BET_), band-gap energy values, and color of TiO_2_ nanoribbons derived from hydrogen titanate nanoribbons under different transformation conditions.

sample	N content^a^ (wt %)	*S*_BET_ (m^2^·g^−1^)	band gap (eV)	color

**TO-400**	—	31	3.50	white
**TO-580**	—	23	3.50	white
**TO-650**	—	21	3.30	white

**TN-400**	0.8	31	3.44	pale yellow
**TN-580**	1.3	32	3.15	yellowish green
**TN-650**	2.5	32	3.10	grey

**CH-W**	—	64	3.49	white
**CH-N**	0.6	45	3.51	white

**MW-W**	—	61	3.52	white

**MW-W+TO**	—	57	3.50	white
**CH-W+TN**	0.3	53	3.52	white

^a^N content was calculated from XPS spectra.

**Figure 7:**
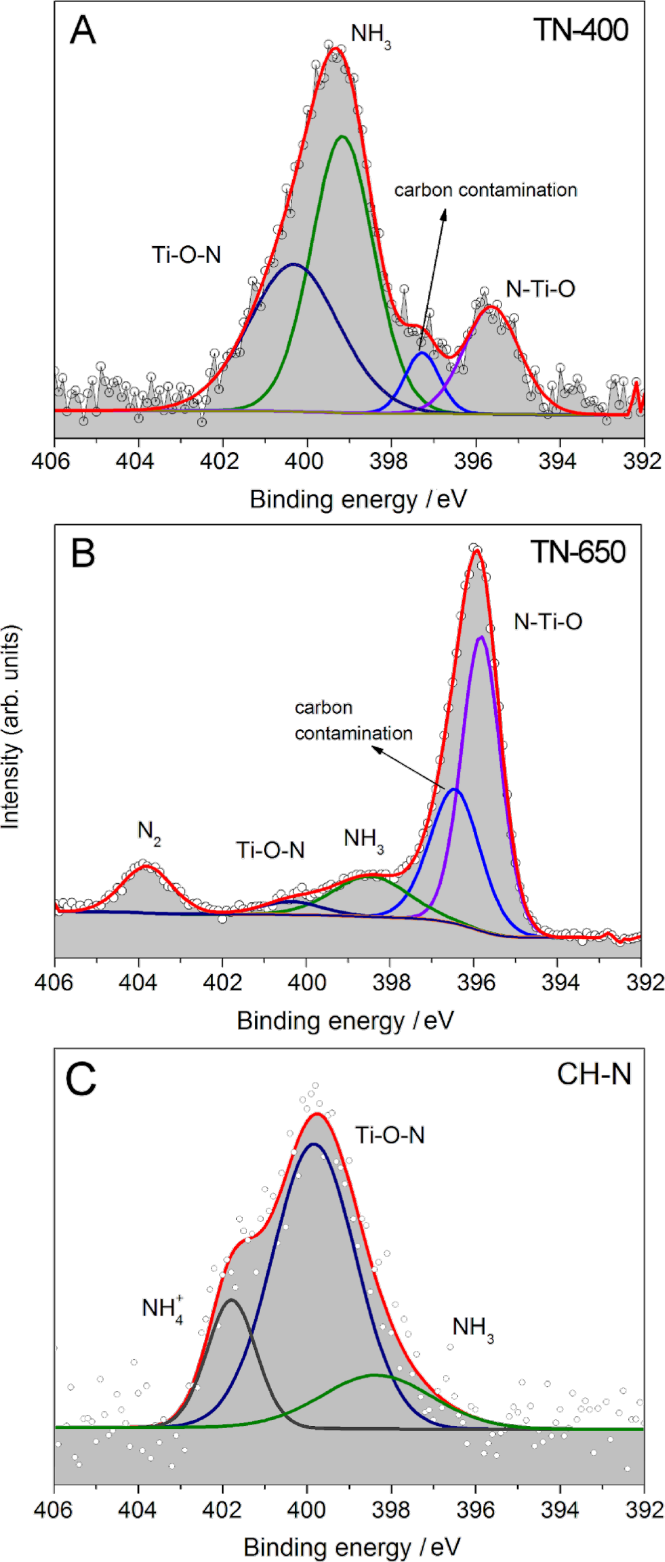
Nitrogen 1s XPS spectra of N-doped TiO_2_ nanoribbons derived from hydrogen titanate nanoribbons resulting from a heat treatment in an NH_3_(g)/Ar(g) flow at 400 °C (A) and 650 °C (B), and hydrothermally in 0.5 M NH_3_(aq) at 160 °C (C).

The significant changes in the color of the N-doped samples (Table 2) are not only due to different N contents; they also indicate the difference in the chemical nature of the nitrogen atoms in the N-doped TiO_2_ NRs (Figure 2). Therefore, we carefully analyzed and compared the N 1s XPS spectra of **TN-400**, **TN-650** and **CH-N** (Figure 7). The most intense peaks in the spectrum of **TN-400** (Figure 7A) are positioned at 400.0 and 400.4 eV, and can be assigned to the adsorbed NH_3_ species and the Ti–O–N linkage, respectively. The Ti–O–N linkage is characteristic for interstitial doping. On the other hand, the peak appearing at 395.6 eV is attributed to the N–Ti–O linkage within TiO_2_, rather than the TiN crystalline phase [32]. As the calcination temperature increases the intensity of the N–Ti–O linkage increases at the expense of the NH_3_ peak and the Ti–O–N linkage (Figure 7A and Figure 7B). Here it should be stressed that the N–Ti–O linkage is characteristic for substitutional N doping, which was reported to be due to the fact that the nitrogen atom of the NH_3_ molecule is “indirectly” bonded to three Ti atoms and thus replaces lattice oxygen in the TiO_2_ matrix [33]. In contrast to **TN-400** and **TN-650** (Figure 7A and Figure 7B), in the N 1s XPS spectrum of **CH-N** (Figure 7C) there is a clear, asymmetric, broadened peak, centered at approx. 400 eV. By fitting the experimental spectrum profile, three different peaks positioned at 397.9, 399.8 and 401.9 eV were identified. The peaks positioned at 397.9 and 401.7 eV are attributed to nitrogen containing species [34] adsorbed onto the surface of the TiO_2_, such as NH_3_ and NH_4_^+^. The most intense peak at 399.8 eV is ascribed to oxidized nitrogen, i.e., Ti–O–N species [35].

Actually, the amount of chemically bonded nitrogen in **TN-580** and **TN-650** is slightly lower than the overall nitrogen content given in Table 2. This is due to trapped elemental nitrogen at the surface (Figure 7B) which is formed by thermal decomposition of NH_3_(g) to N_2_(g) and H_2_(g) at temperatures above 550 °C [36]. In the samples calcined at 580 °C and 650 °C 20 and 10 wt %, respectively, of the overall nitrogen is in the form of N_2_. In **TN-400** no N_2_ was observed [36] (Figure 7A). Additionally, a comparison of the nitrogen content (Table S1, Supporting Information File 1) in the TiO_2_ NRs thermally treated at 400 °C in an ammonia atmosphere derived from three different precursors, **HTiNRs**, **TO-580** and **CH-W,** revealed that the best precursor for N doping, with respect to the N content, is hydrogen titanate.

The effect of nitrogen substitutional doping should also be reflected in the position of the Ti 2p XPS peak. A small energy shift of the Ti 2p peak to lower binding energies is observed as the calcination temperature is increased (Figure S6, Supporting Information File 1). This can be attributed to the interaction between titanium and nitrogen atoms replacing the interaction between titanium and oxygen atoms [32,37–39]. In the titanium–nitrogen bonding there are fewer electrons participating and therefore the screening of the titanium nucleus is smaller than in the case of the titanium–oxygen bonding [40]. The observed energy shift indicates that the N atoms are incorporated into the lattice and they substitute the oxygen atoms. In fact, we observe an increase in the relative area of the peak corresponding to the formation of the N–Ti–O linkage with an increased calcination temperature: **TN-400** (0.2%), **TN-580** (0.6%) and **TN-650** (2.5%). This finding is also in agreement with the presence of the titanium oxynitride phase observed in **TN-650** (Figure 2). Interestingly, no Ti^3+^ was observed in any of the Ti 2p XPS spectra of the N-doped TiO_2_ NRs.

### Characterization of paramagnetic centers with EPR spectroscopy

**Figure 8:**
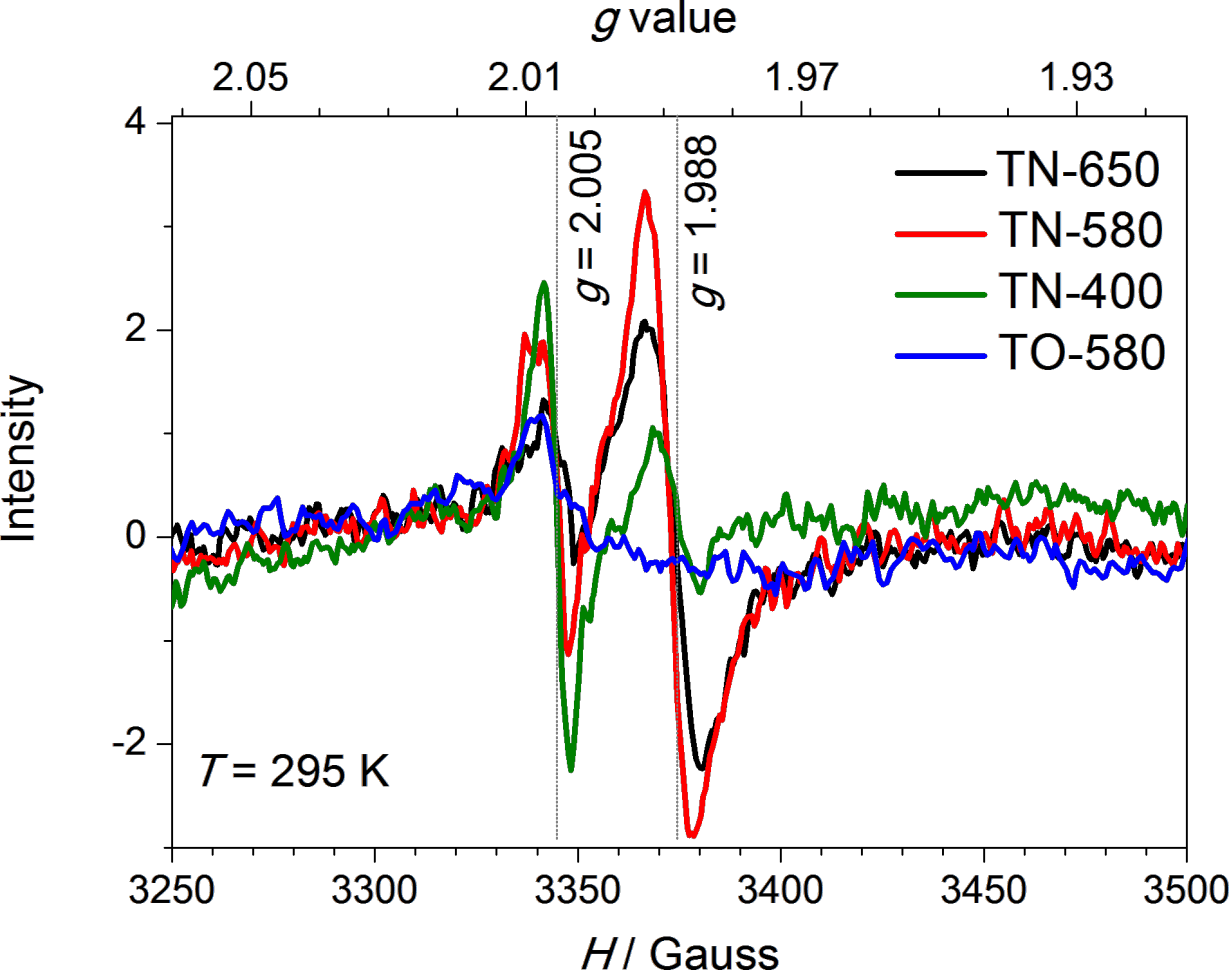
Room-temperature EPR spectra of TiO_2_ nanoribbons derived from hydrogen titanate nanoribbons by calcination in an NH_3_(g)/Ar(g) flow (**TN-650**, **TN-580** and **TN-400**) and air (**TO-580**).

Ammonia gas has the ability to reduce Ti^4+^ in TiO_2_ to Ti^3+^, or even Ti^2+^. In our case, the XPS only showed the presence of Ti^4+^ in the N-doped TiO_2_ NRs. In order to elucidate this, a local-probe technique with much higher sensitivity, i.e., electron paramagnetic resonance (EPR) spectroscopy was employed. The room temperature EPR spectra of the samples treated in the NH_3_(g)/Ar(g) flow, and in the atmosphere of air are shown in Figure 8. In all cases, a resonance at *g* ≈ 2.005 is observed, which is, in the N-doped TiO_2_ samples, typically related to oxygen vacancies [35,41]. In addition to this resonance, in the samples that were treated in reductive atmosphere, i.e., in **TN-400**, **TN-580** and **TN-650**, exhibit another intense line at *g* ≈ 1.988, which is assigned to the Ti^3+^ centers in the bulk. Moreover, as the reaction temperature increases the intensity of this resonance line for **TN-400** and **TN-580** also increases. On the contrary, the intensity of the Ti^3+^ EPR signal in **TN-650** is markedly lower although, more Ti^3+^ is expected in this sample due to the presence of TiON phase (Figure 2). A similar effect was observed by Zhang et al. [40] and Pan et al. [42], and a tentative explanation involves high charge mobility.

**Figure 9:**
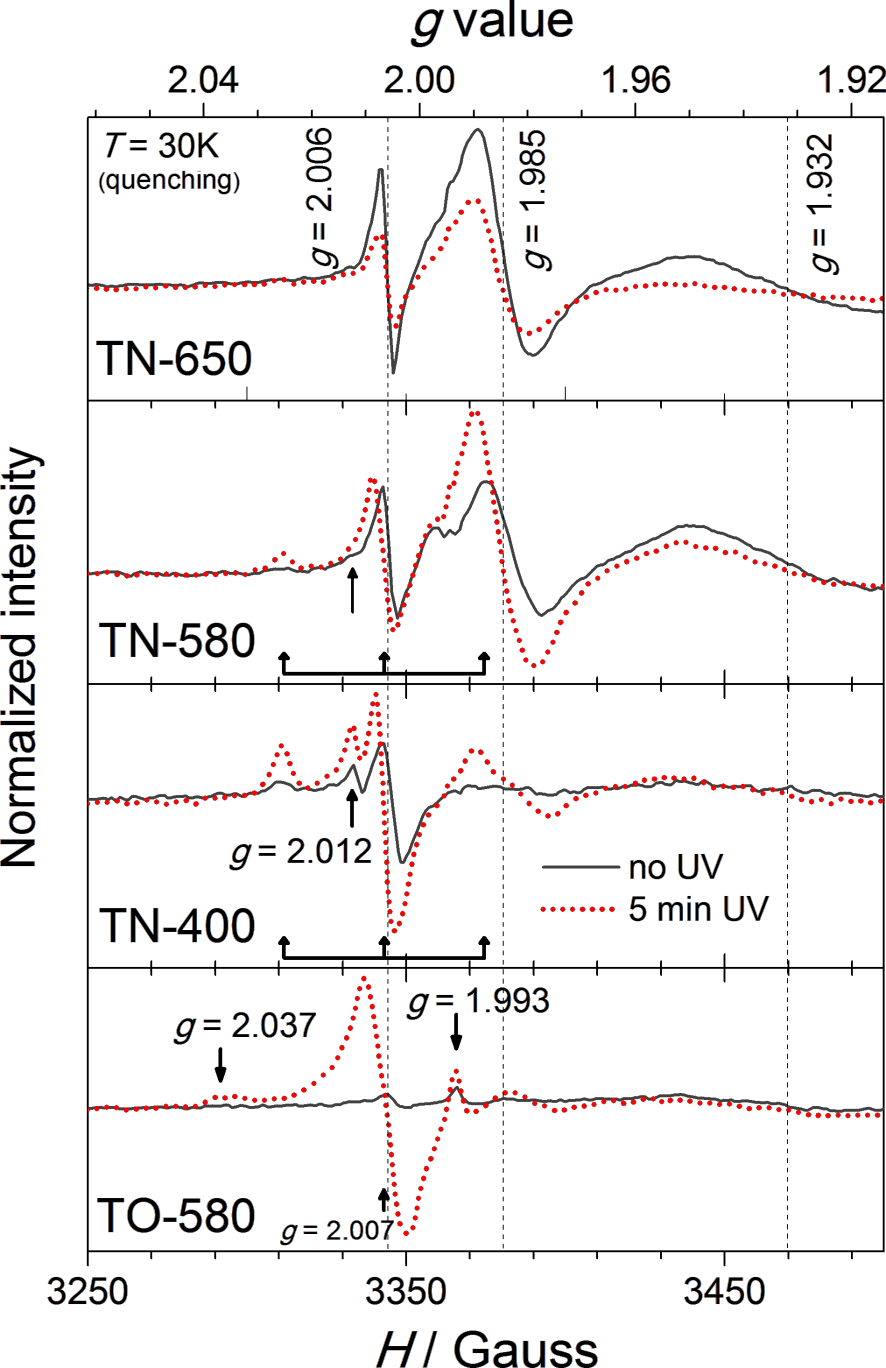
EPR spectra of TiO_2_ nanoribbons derived from hydrogen titanate nanoribbons by calcination in an NH_3_(g)/Ar(g) flow (**TN-650**, **TN-580** and **TN-400**) and air (**TO-580**) measured in darkness (grey lines) and after 5 min of UV–vis illumination (red dotted lines). Samples were quenched from room temperature to 30 K.

Additionally, we characterized paramagnetic centers of the selected TiO_2_ NRs created under UV–vis illumination (Figure 9). To ensure sufficient life span of the excited paramagnetic species, the samples were quenched from room temperature to 30 K, and then measured in darkness (gray spectral lines) and after 5 min of UV–vis illumination (red dotted spectral lines). We note that longer illumination times did not change the EPR spectra significantly, i.e., the excited species had been already saturated. For samples that were calcined below 600 °C, i.e., **TO-580**, **TN-400** and **TN-580**, both resonances that were observed at room temperature (Figure 8) increase, i.e., the number of oxygen vacancies and the Ti^3+^ ions increases. Moreover, the former are most likely responsible also for the resonances appearing at *g* values [35,41] of approx. 2.012, and approx. 2.037 (marked with arrows in Figure 9) that partly overlap with the triplet signal of N-containing paramagnetic species [40,43] centered at a *g* value of approx. 2.007 (marked with a triple arrow). The effect is the most pronounced for **TO-580**. An additional signal with the *g* value of approx. 1.993 appears in the spectrum of **TO-580**. Most probably it arises from Ti^3+^ in the bulk, which is slightly shifted when compared to the N-doped samples. This shift reflects the different chemical environment of Ti^3+^. In the case of **TN-650** the opposite effect is observed, the intensity of the spectrum decreased after the illumination. A plausible explanation for the observed effect is the same as for the spectrum measured at room temperature, i.e., this sample is more conductive than the others and thus more prone to the charge mobility, which is enhanced by illumination, leading to a reduction of the EPR signal.

### Specific surface area

As revealed by the microscopy investigations (Figure 4, Figure 5, Figure 6 and Figure S5, Supporting Information File 1) the different transformation environments affect the surfaces of the nanoribbons. Therefore, the specific surface area of the selected samples was evaluated (Table 2). In general, for the samples calcined in air a decrease in the specific surface area is observed. As the calcination temperature is increased from 400 to 650 °C, the specific surface area is reduced by about one-third, from 31 to 21 m^2^/g. Surprisingly, this trend is not observed for the samples calcined in the NH_3_(g)/Ar(g) flow. In this case the specific surface area remains constant (approximately 31 m^2^/g). On the other hand, the hydrothermal conditions during the transformation of HTiNRs to TiO_2_ (**CH-W**, **CH-N** and **MW-W**) significantly affected the surfaces of nanoribbons (Figure 5 and Figure 6), which is reflected in the increase of specific surface area, i.e., by a factor of about 3 compared to **TO-650**. As expected, an additional calcination of **CH-W** and **MW-W** caused a slight decrease in the specific surface area of **CH-W+TN** and **MW-W+TO**.

### Optical band-gap features

The band gap of the precursor HTiNRs is 3.65 eV and, as expected, the transformation to TiO_2_ NRs resulted in a narrowing of this band gap. Table 2 summarizes the band gaps of the selected TiO_2_ NR samples. In the set of samples calcined in air the band gap narrowed by about 0.2 eV. A slightly more pronounced effect is observed for the set of samples calcined in the NH_3_(g)/Ar(g) flow. In this case the band gap narrowed by about 0.3 eV when the calcination temperature was increased from 400 to 650 °C. This is attributed to the increasing amount of N–Ti–O linkage [23] and is fully in line with the XPS results (Figure 7A and Figure 7B). The band-gap values for the anatase NRs derived under the hydrothermal and microwave-assisted hydrothermal conditions in different transformation environments do not differ significantly with respect to each other.

The doping of TiO_2_ with N is known to shift the absorbance toward visible light [23], compared with the undoped TiO_2_. The optical absorption spectrum of **TN-580** (Figure S7, see Supporting Information File 1) shows that the sample absorbs light up to 500 nm, whereas **TO-580** and **CH-W** do not. A comparison of the diffuse reflectance spectra of the N-doped TiO_2_ (not shown) reveals that with the increased reaction temperature, which is reflected in higher N content, the transparency of the obtained N-doped TiO_2_ NRs is decreased in the visible part of the measured region. The same effect was reported by Zhang et al. [40]. This is explained with the creation of new extra states within the band-gap states created by substitutional and interstitional N-doping [23,36] and to creation of an additional localized state due to oxygen vacancies below the conduction band [36].

### Assessment of the photocatalytic performance of the TiO_2_ NRs

The photocatalytic performance (PP) of the prepared TiO_2_ NR samples was evaluated by monitoring the photocatalytic oxidation of isopropanol (iPrOH), for which acetone is the main product. For the purposes of this study, the mechanism of iPrOH oxidation is simplified in accordance with the following two-step reaction (Equation 1):

[1]
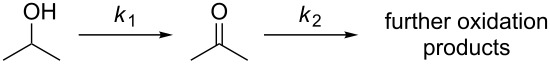


The first reaction is considered to be a zero-order process, while the second one is a first-order reaction. The mechanism of the photocatalytic oxidation of iPrOH under UV–vis light is explained in detail by Marolt et al. [44].

**Figure 10:**
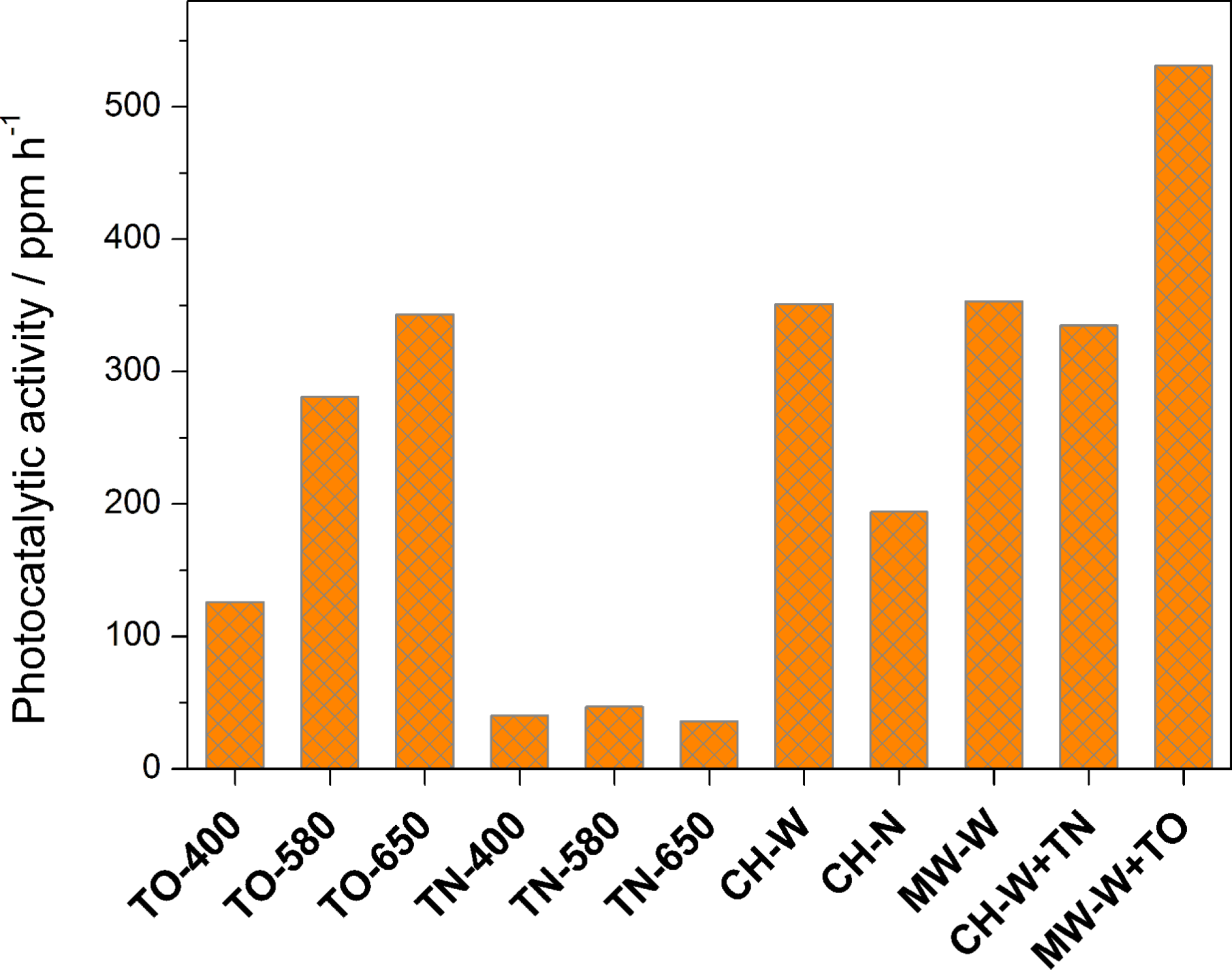
Comparison of the photocatalytic performance under UV–vis light for a series of TiO_2_ nanoribbon samples.

A comparison of the PP for different TiO_2_ NR samples is presented in Figure 10. In the first set of samples calcined in air, despite an approximately 30% decrease in the specific surface area (Table 2), a two-fold increase in the PP is observed as the calcination temperature increases from 400 to 650 °C. This considerable improvement can be explained (i) by the increasing amount of anatase and (ii) the improved crystallinity (Figure 1). Meanwhile, the estimated PP of the samples calcined in the NH_3_(g)/Ar(g) flow is approximately the same (ca. 40 ppm/h), and it is drastically reduced compared with the samples calcined in air, although the content of anatase phase increases with an increasing calcination temperature (Figure 2). The observed effect is in agreement with the findings reported by Irie et al. [36], and Miyauchi et al. [45]. In a similar system, i.e., the photo-oxidation of iPrOH, they noticed that with an increasing N content, longer exposure times to UV and visible light were required to decompose the iPrOH. In our case this is reflected in lower *k*_1_ values [44]. The calcination of the TiO_2_ in an ammonia atmosphere increases the amount of oxygen vacancies and Ti^3+^, which is in agreement with the EPR results (Figure 8 and Figure 9). The oxygen vacancies act as recombination centers for the holes and electrons, and are therefore responsible for the decrease in the PP of the N-doped TiO_2_ [36].

The PP of the samples converted to TiO_2_ NRs hydrothermally (i) by convective heating in an autoclave and (ii) microwave-accelerated heating in a microwave reactor (**CH-W** and **MW-W**) is approximately the same as for **TO-650** (ca. 345 ppm/h). Most likely, the increased specific surface area of these samples (Table 2) contributed a great deal to their higher PP values. Again, the presence of N significantly affected the PP of the **CH-N** sample, which was reduced by half compared to the PP of **CH-W** and **MW-W**.

The specific surface area and the crystallinity are two of the key factors that strongly affect the photocatalytic activity of TiO_2_. Therefore, with the aim to improve the crystallinity of the TiO_2_ NRs the samples with the highest specific surface area (**CH-W** and **MW-W**) were additionally calcined in air and in the NH_3_(g)/Ar(g) flow (**MW-W+TO**, **CH-W+TN**). As expected, the PP of **MW-W+TO** increased by about 50% (Figure 10) with respect to the starting material (**MW-W**). Even when the additional thermal treatment was made in the reductive atmosphere, only a slight decrease in the PP was observed. The reason for such a small decrease in the PP is the relatively small amount of N (ca. 0.3 wt %, Table 2). In addition, the PP activities of the TiO_2_ NRs samples were compared to the PP of Degussa P25, which is 604 ppm/h.

## Conclusion

In this study we used hydrogen titanate nanoribbons as a precursor material for the preparation of TiO_2_ nanoribbons. By a careful selection of the transformation strategy and the reaction parameters for the transformation of H_2_Ti_3_O_7_ to TiO_2_ we were able to affect the phase composition and the surface of the prepared TiO_2_ nanoribbons. The phase composition was tuned with the reaction temperature during the heat treatment in air or an NH_3_(g)/Ar(g) flow. These conditions promoted the titanate transformation to anatase via a metastable TiO_2_-B phase. Conversely, when the transformations proceeded hydrothermally, a direct transformation from H_2_Ti_3_O_7_ to anatase TiO_2_ was observed. Furthermore, during the hydrothermally induced transformation on the surface of the TiO_2_ nanoribbons small nanoparticles were formed and their presence considerably increased the specific the surface area of the TiO_2_ nanoribbons. Furthermore, the shape of these particles was influenced by the pH of the reaction environment. The heating of HTiNRs in an NH_3_(g)/Ar(g) flow or NH_3_(aq) resulted in the N doping of the TiO_2_ nanoribbons where the N content was tuned with the calcination temperature and/or the choice of the precursor. Interestingly, the hydrothermally induced transformation in NH_3_(aq) resulted in interstitial doping only, while interstitial and substituitonal doping were result of calcination of HTiNRs in the NH_3_(g)/Ar(g) flow.

The photocatalytic efficiency of TiO_2_ depends on the properties of the particles and on the target organic molecules. In our case the photocatalytic performance was studied by using the photo-oxidation of isopropanol under UV–vis light as the model reaction. The photocatalytic performance of the TiO_2_ nanoribbons calcined in air continuously increased with the increasing calcination temperature and the anatase content. The presence of N as a dopant in the TiO_2_ matrix significantly reduced the photoactivity of the TiO_2_ nanoribbons, regardless of the doping method. This suggests that adsorption properties of ispropanol to the surface of the N-doped TiO_2_ nanoribbons were significnalty reduced in comparison with the undoped TiO_2_ nanoribbons. A significant improvement in the photocatalytic performance was achieved by combining a hydrothermally induced transformation, which resulted in an increase of specific surface area, and an additional calcination in air which improved the crystallinity of the TiO_2_ nanoribbons.

## Experimental

### Synthesis

#### Preparation of hydrogen titanate nanoribbons

Hydrogen titanate nanoribbons (HTiNRs) were prepared from sodium titanate nanoribbons [25] (NaTiNRs) by an ion exchange. In brief, a suspension of 2.0 g of NaTiNRs and 150 mL of 0.1 M CH_3_COOH(aq) was stirred for 15 min and centrifuged. This procedure was repeated five times. After the last centrifugation the sediment was washed first with deionized water until the pH of the supernatant was ca. 5.5, then rinsed with ethanol (EtOH), and finally dried in an oven at 100 °C for 12 h.

#### Transformation of hydrogen titanate nanoribbons to TiO_2_ nanoribbons

**Heating in static air:** In general, 150 mg of HTiNRs was weighed in an alumina boat, placed into an oven and heated at a ramp rate of 1 °C/min to 400, 580 or 650 °C. The samples were kept at the selected temperatures for 6 h, and cooled down to room temperature afterwards. The samples were labeled **TO-400**, **TO-580** and **TO-650**, where the number refers to the calcination temperature.

**Heating in an NH_3_(g)/Ar(g) flow:** HTiNRs (225 mg) were weighed in a quartz boat, placed into an oven, heated at a ramp rate of 7.2 °C/min to 400, 580 or 650 °C. The samples were kept at the selected temperatures for 6 h, and cooled down to room temperature afterwards. The flow ratio of NH_3_(g)/Ar(g) was 30 mL·min^−1^/10 mL·min^−1^, respectively. The samples were labeled **TN-400**, **TN-580** and **TN-650**, where the number refers to the calcination temperature.

**Convective heating in a hydrothermal reactor:** HTiNRs (150 mg) were suspended in either 36 mL of deionized water or 0.5 M NH_3_(aq). The prepared reaction mixture was placed into a Teflon-lined hydrothermal reactor (Berghof, BR25, filling volume was 80%) and heated at a ramp rate of 1 °C/min to 160 °C at a constant stirring speed of 300 rpm for either 10 or 24 h. After being cooled down to room temperature the product mixture was centrifuged then washed with EtOH, and finally dried in an oven at 100 °C for 12 h. The samples were labeled **CH-W** and **CH-N**, where the last letter indicates the reaction medium, water (W) or 0.5 M NH_3_(aq) (N), in which the transformation occurred.

**Microwave-assisted treatment:** HTiNRs (150 mg) were suspended in 20 mL of deionized water. The prepared reaction mixture was transferred in a 30 mL glass vial and inserted into a microwave reactor and heated at a constant temperature of 200 °C for two hours under constant stirring (300 rpm). After being cooled down to room temperature the product mixture was first centrifuged then washed with EtOH and finally dried in an oven at 100 °C for 12 h. The sample was labeled **MW-W**. Microwave-assisted transformations took place in an Anton Paar microwave reactor Monowave 300.

**Additional heat treatment:** Two samples, **CH-W** and **MW-W** (150 mg), were additionally calcined for 6 h in static air or a dynamic NH_3_(g)/Ar(g) atmosphere at 480 °C and 400 °C, respectively. A flow ratio of NH_3_(g)/Ar(g) was set to 30 mL·min^−1^/10 mL·min^−1^. Derived samples were labeled **CH-W+TO** and **MW-W+TN**.

#### Materials characterization

The morphology of the products was investigated with scanning (FE-SEM, Jeol 7600F) and transmission electron microscopes (TEM Jeol 2100, 200 keV). For the SEM analysis the samples were dispersed in water and the drop of dispersion was deposited on a polished Al sample holder. Prior to the SEM investigation an about 3 nm thick carbon layer was deposited on the samples to reduce the charging effect. Specimens for TEM investigations were dispersed ultrasonically in methanol and a drop of the dispersion was deposited onto a lacy carbon film supported by a copper grid.

The phase analysis was performed on the cut surface by X-ray powder diffraction (XRD) by using a diffractometer with Cu Kα radiation (λ = 1.5406 Å) and a Sol-X energy-dispersive detector (Endeavor D4, Bruker AXS, Karlsruhe, Germany). Diffractograms were measured in the angular range (2θ) between 5 and 60° with a step size of 0.02 °/s, and a collection time of 3 s.

For the determination of the nitrogen content X-ray photoelectron spectroscopy (XPS) measurements were performed with a VERSAPROBE PHI 5000 from Physical Electronics, equipped with a monochromatic Al Kα X-ray source. The energy resolution was 0.6 eV. A dual beam charge neutralizator composed of an electron gun (ca. 1eV) and an Argon Ion gun (≤ 10eV) was used in order to compensate the built-up charge on the surface of the specimens during the measurements. The specimens for the XPS measurements were prepared by pressing the specimen into a pellet. A conductive double-sided tape was used to attach the pellet to a sample holder.

Diffuse reflectance spectra were acquired in the 200–800 nm range with a 0.5 nm step size using a UV–vis–NIR spectrometer (Shimadzu UV-3600) equipped with an integrating sphere (ISR-3100, 60 mm) and BaSO_4_ as a standard. The Kubelka–Munk function was applied to convert the diffuse reflectance into the absorbance [46]. The optical band-gaps energy (*E*_g_) was determined from the wavelength at which the tangent of the absorbance line intersected the abscissa coordinate.

The chemical analysis of the sodium content was done using a FE-SEM equipped with an energy dispersive X-ray spectrometry (EDX) elemental analysis system. The samples for the EDX measurements were prepared by pressing the powder samples into pellets and coating them with a thin carbon layer.

The BET specific surface areas of the samples were measured at −196 °C with a TRISTAR 3000 automated gas-adsorption analyzer.

Continuous wave EPR measurements were performed at room temperature in X-band (frequency about 9.4 GHz) with a custom-built spectrometer, which was equipped with a Varian E-101 microwave bridge. Approximately 5 mg of the samples were weighed in the EPR tubes in order to quantitatively compare the measured EPR intensities. The EPR tubes were then vacuumed and sealed. EPR spectra were first measured at room temperature and then the samples were quenched to 30 K where spectra were first measured in the dark and then after 5 min of UV–vis illumination. A mercury lamp was used for in situ UV–vis light illumination of the samples in an EPR cavity. An Oxford Cryogenics continuous-flow liquid He cryogenic system ensured a temperature stability with a range of fluctuation below 0.1 K.

The thermogravimetric (TG) measurements were performed on a Mettler Toledo TGA/DSC 1 Instrument from room temperature up to 800 °C with a heating rate of 10 K/min. Samples with an initial mass of around 10 mg were placed into 150 µL Pt crucibles. During the measurement, the furnace was purged with an air flow with a rate of 50 mL·min^−1^. The baseline was automatically subtracted from the measured TGA curve. Differential scanning calorimetry (DSC) measurements were performed separately on a Mettler Toledo DSC 1 Instrument. Around 5 mg of samples was weighed into 70 µL/min Pt crucibles; empty crucibles were used as a reference. The upper temperature of the measurements was 700 °C. Other parameters (heating rate, purge gas) were identical to those used for the TG measurements.

#### Measurements of photochemical performance

The photocatalytic performance of the prepared TiO_2_ NRs was measured in a sealed gas–solid continuous flow reactor system. The concentrations of isopropanol (iPrOH), as the model pollutant, and acetone, the first product of iPrOH oxidation, were continually monitored with FTIR spectroscopy. The method is described in detail in [44,47]. In brief, 50 mg of sample was evenly spread on a petri dish, 60 mm in diameter, and put into the system. Then, 8 µL of iPrOH, corresponding to about 800 ppm, was injected in the system. After the adsorption–desorption equilibrium was reached (Figure S8, Supporting Information File 1), as indicated by the flat lines of the iPrOH and acetone concentration, the sample was illuminated. When the light was switched on the iPrOH concentration began to fall and the acetone concentration began to rise. The system was kept at a temperature of 25 ± 3 °C and a relative humidity of 22 ± 3%.

The light source was a 300 W Xe lamp (Newport Oriel Instrument, USA). The lamp imitates the solar spectrum and emits both ultraviolet (UV) and visible light (vis). The radiance intensity on the sample was 40 W/m^2^. The iPrOH and acetone concentrations were calculated by observing characteristic peaks at 951 cm^−1^ and 1207 cm^−1^, respectively, using a FTIR spectrometer (Perkin Elmer BX II).

## Supporting Information

File 1 SEM and TGA and DSC data for the precursor hydrogen titanate nanoribbons sample, XRD patterns, SEM and TEM images, XPS and optical absorbance spectra of selected TiO_2_ nanoribbon samples, and concentration profiles of isopropanol and acetone during the photocatalytic oxidation of isopropanol under UV–vis illumination.
