# Supplementary material for: Transformation of hydrogen titanate nanoribbons to TiO2 nanoribbons and the influence of the transformation strategies on the photocatalytic performance
Source: Beilstein J Nanotechnol. 2015 Mar 27;6:831–44. doi: 10.3762/bjnano.6.86 (PMC4419672; doi:10.3762/bjnano.6.86)
Supplement: File 1 — SEM and TGA and DSC data for the precursor hydrogen titanate nanoribbons sample, XRD patterns, SEM and TEM images, XPS and optical absorbance spectra of selected TiO2 nanoribbon samples, and concentration profiles of isopropanol and acetone during the photocatalytic oxidation of isopropanol under UV–vis illumination. [file Beilstein_J_Nanotechnol-06-831-s001.pdf]

Supporting Information

for

Transformation of hydrogen titanate nanoribbons to TiO_2 nanoribbons and the influence of the transformation strategies on the photocatalytic performance

Melita Rutar[1,2], Nejc Rozman[3], Matej Pregelj[1], Carla Bittencourt[4], Romana Cerc Korošec[5], Andrijana Sever Škapin[3], Aleš Mrzel[1], Srečo D. Škapin[1] and Polona Umek*[1]

Address: [1]Jožef Stefan Institute, Jamova cesta 39, SI-1000 Ljubljana, Slovenia; [2]Jožef Stefan International Postgraduate School, Jamova cesta 39, SI-1000 Ljubljana, Slovenia; [3]Slovenian National Building and Civil Engineering Institute, Dimičeva 12, SI-1000 Ljubljana, Slovenia; [4]Chimie des Interactions Plasma Surface, CIRMAP, University of Mons, 23 Place du Parc, B-7000 Mons, Belgium and [5]Faculty of Chemistry and Chemical Technology, University of Ljubljana, Aškerčeva cesta 5, SI-1000 Ljubljana, Slovenia

Email: Polona Umek - polona.umek@ijs.si

*Corresponding author

Additional experimental data

Characterization of precursor hydrogen titanate nanoribbons

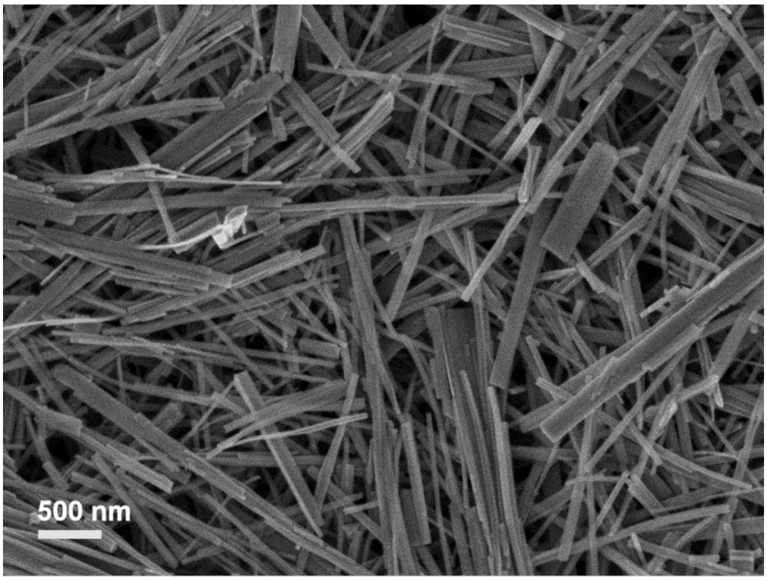

Figure S1: SEM image of precursor hydrogen titanate nanoribbons.

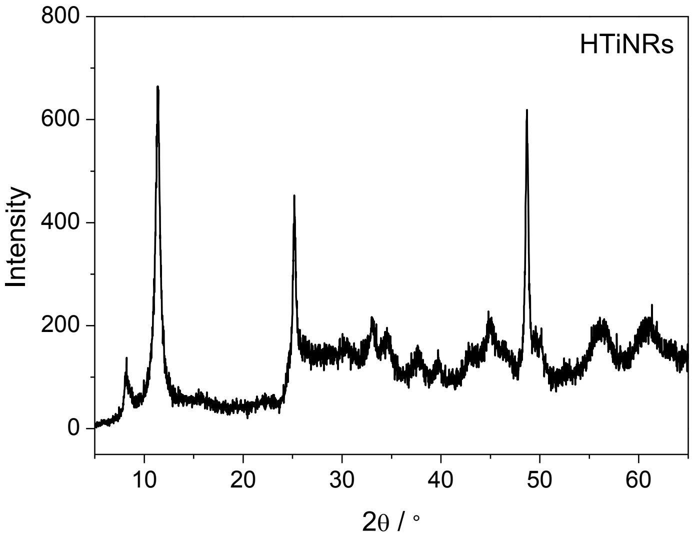

Figure S2: Characteristic XRD pattern of precursor hydrogen titanate nanoribbons.

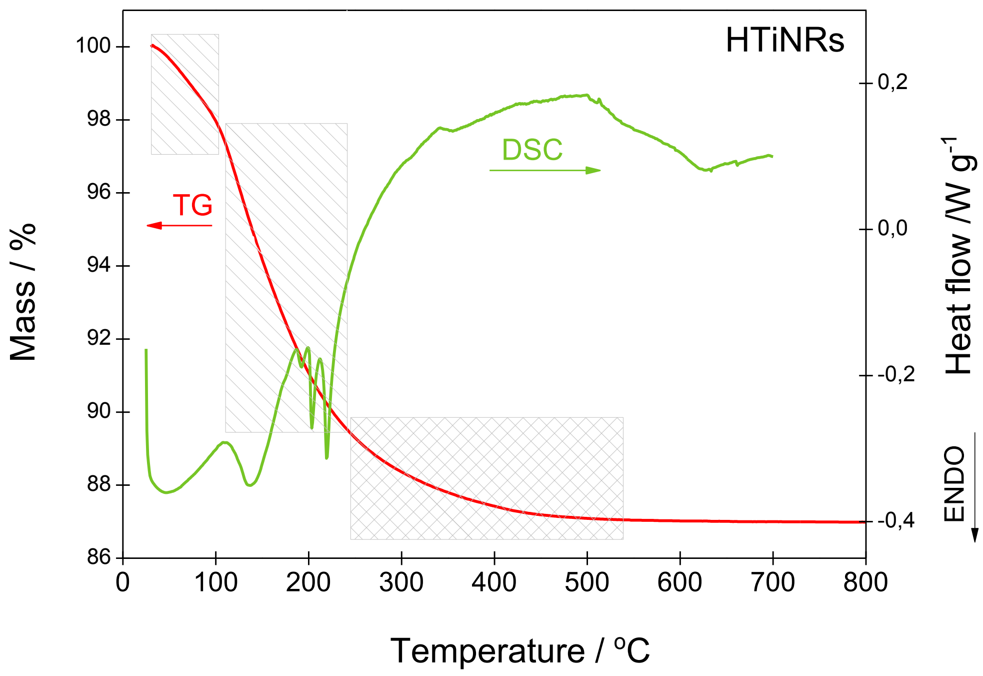

Figure S3: TGA (red) and DSC (green) curves of precursor hydrogen titanate nanoribbons.

XRD Patterns, TEM and SEM images, XPS and optical absorbance spectra of selected TiO_2 nanoribbon samples

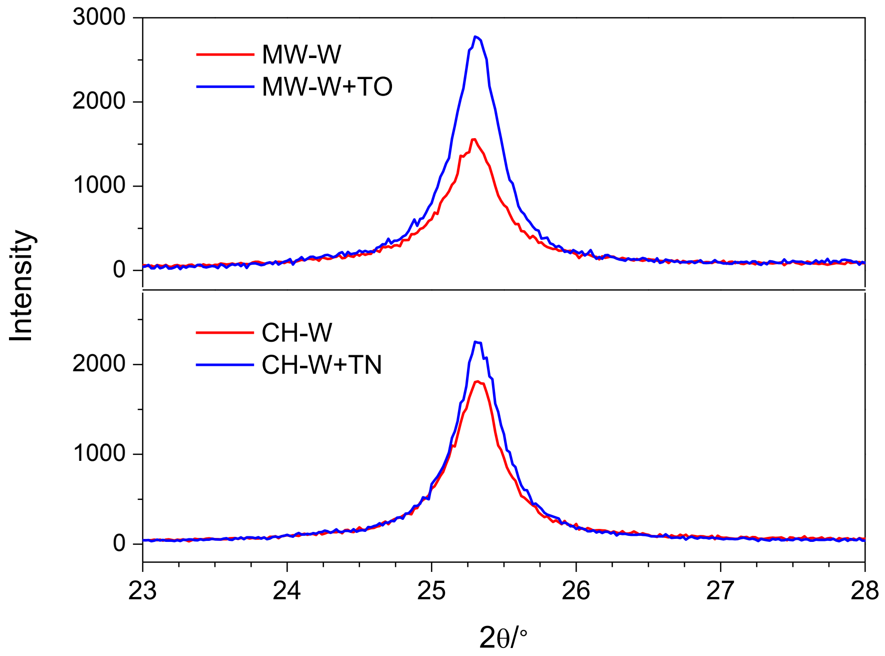

Figure S4: Expanded views of the anatase(101) peak of **MW-W+TO** (top) and **CH-W+TN** (bottom) compared to **MW-W** and **CH-W**, respectively.

Values of the full width at the half maximum (FWHM) of the anatase(101) peak of **MW-W-TO** and **CH-W-TN** narrowed by about 0.1° when compared to **MW-W** and **CH-W**. Thus, it can be concluded that the additional calcination in air or the NH_3(g)/Ar(g) flow after the hydrothermal treatment slightly improves the crystallinity of the material.

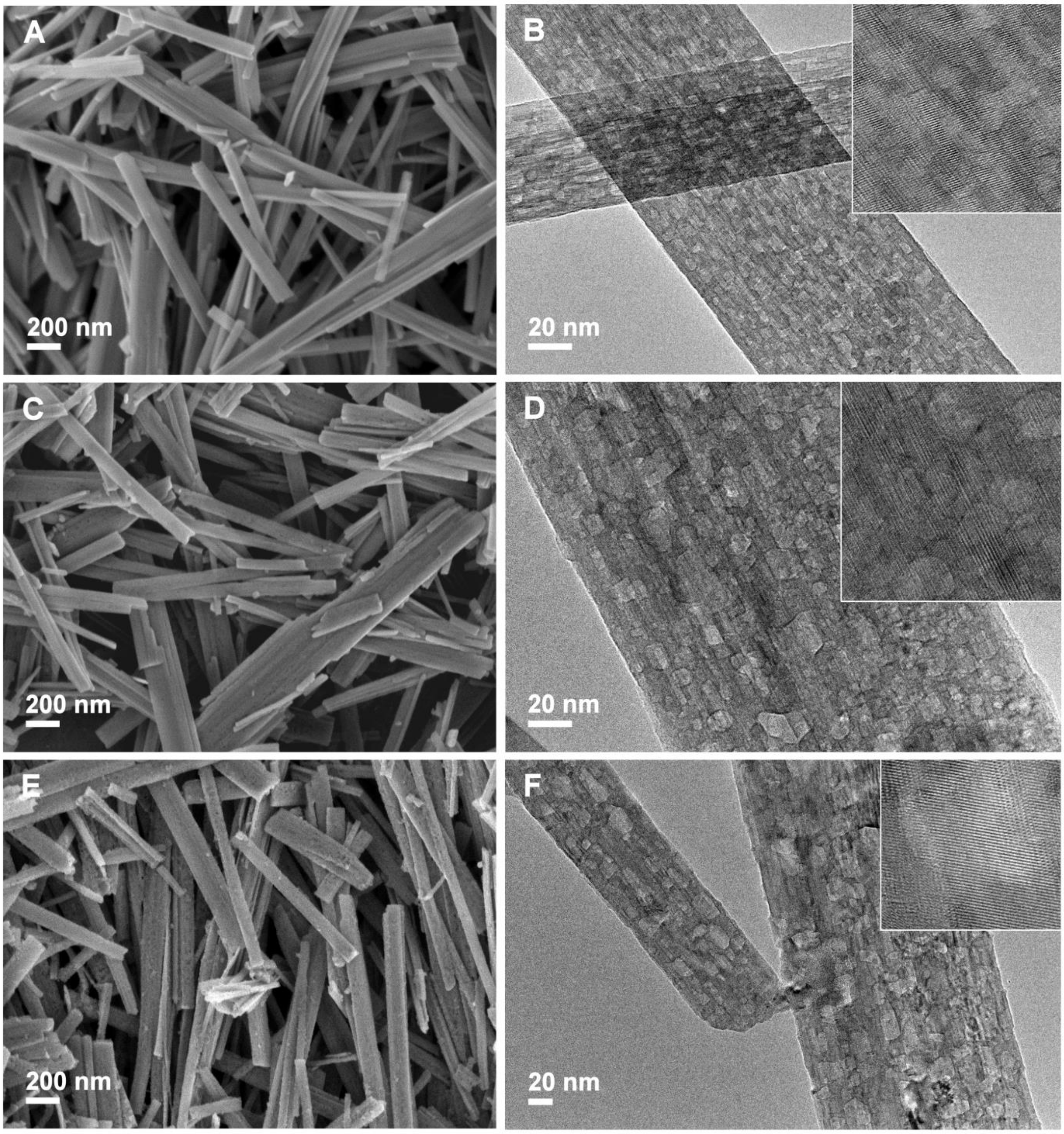

Figure S5: SEM and TEM images of TiO_2 nanoribbons derived from hydrogen titanate nanoribbons by heat treatment in an NH_3(g)/Ar(g) flow at 400 °C (A and B), 580 °C (C and D), and 650 °C (E and F). The insets in figures B; D and F indicate the crystalline nature of the nanoribbons.

Table S1: Calcination temperature in an NH_3(g)/Ar(g) flow and N content for N-doped TiO_2 nanoribbons prepared from different precursors.

sample	precursor	*T* (°C)[a]	N content (wt %)[b]
**TN-400**	HTiNRs	400	0.8
**TN-580**	HTiNRs	580	1.3
**CH-W+TN-400**	CH-W	400	0.3.
**CH-W+TN-580**	CH-W	580	0.4
**TO-580+TN**	TO-580	400	<0.1

[a]The ramp time at selected temperature was 6 h.

[b]N content was determined by XPS.

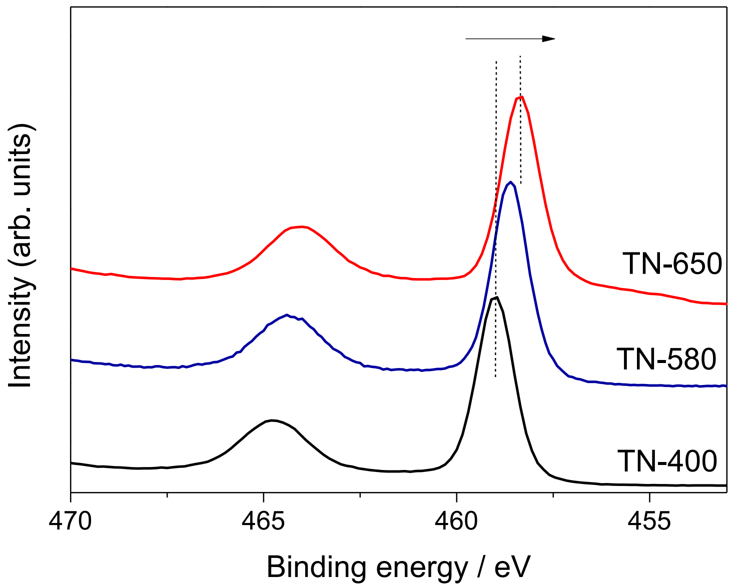

Figure S6: Ti 2p XPS spectra of N-doped TiO_2 nanoribbons derived from hydrogen titanate nanoribbons resulting from calcination in an NH_3(g)/Ar(g) flow at 400 °C (**TN-400**), 580 °C (**TN-580**), and 650 °C (**TN-650**). The relative amount of the O–Ti–N linkage in these samples increases with the calcination temperature (0.22%, 0.65% and 2.50%), respectively. Two dotted lines show a shift of the Ti 2p peak to lower binding energies with increased calcination temperature.

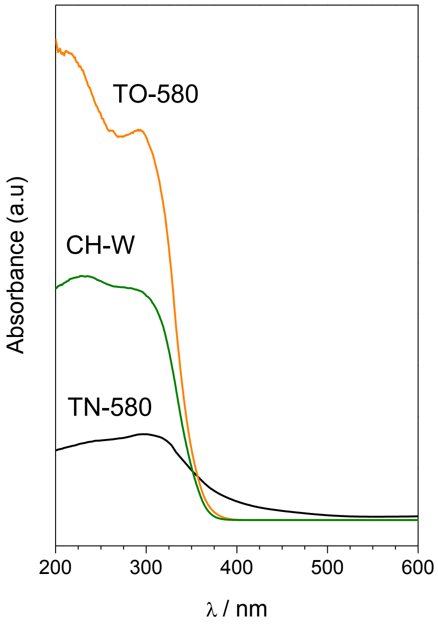

Figure S7: Optical absorbance spectra of TiO_2 nanoribbons derived from hydrogen titanate nanoribbons by calcination in air (**TO-580**), an NH_3(g)/Ar(g) flow (**TN-580**) and hydrothermal treatment (**CH-W**).

Concentration profile of isopropanol and acetone during the photocatalytic oxidation of isopropanol under UV–vis illumination

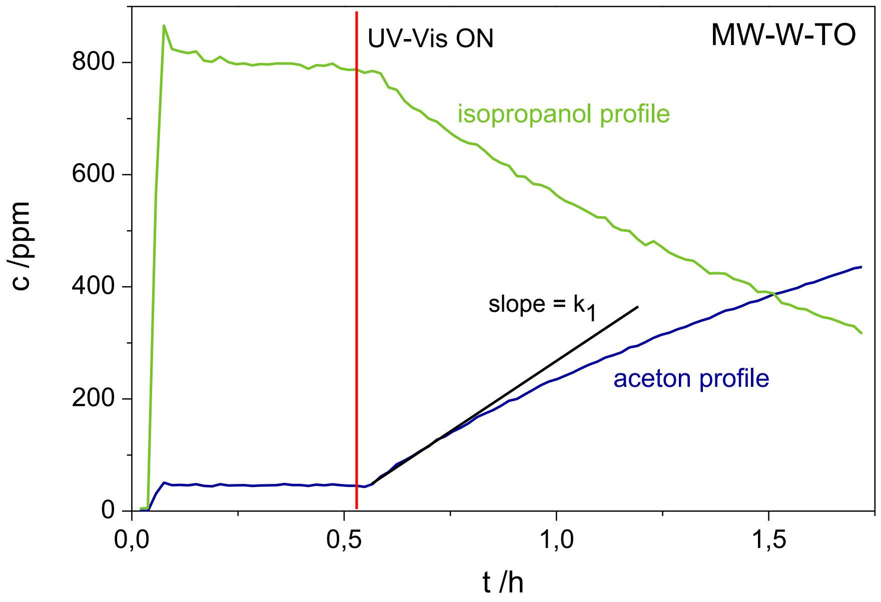

Figure S8: Characteristic curves representing the oxidation of iPrOH under UV–vis illumination catalyzed by **MW-W-TO**; decreasing iPrOH concentration (green) and simultaneous increase of acetone concentration (blue). The red line is positioned at the time when UV–vis light was switched on.

When iPrOH is injected into a reaction chamber an immediate decrease in isopropanol concentration is observed. This is due to the adsorption of iPrOH on the surface of the sample and the surface of the reaction chamber. Switching on the UV–vis light source is reflected in a decrease of iPrOH concentration and a simultaneous increase of acetone concentration. The constant $k_{1,}$ which we used as an assessment of the photocatalytic performance of the samples, was calculated from the initial slope of the acetone profile.
